# Reducing Calibration Bias for Person Fit Assessment by Mixture Model Expansion

**DOI:** 10.1177/00131644251364252

**Published:** 2025-09-06

**Authors:** Johan Braeken, Saskia van Laar

**Affiliations:** 1 University of Oslo, Oslo, Norway; 2 Maastricht University, Maastricht, the Netherlands

**Keywords:** person fit, measurement appropriateness, mixture, random responders

## Abstract

Measurement appropriateness concerns the question of whether the test or survey scale under consideration can provide a valid measure for a specific individual. An aberrant item response pattern would provide internal counterevidence against using the test/scale for this person, whereas a more typical item response pattern would imply a fit of the measure to the person. Traditional approaches, including the popular Lz person fit statistic, are hampered by their two-stage estimation procedure and the fact that the fit for the person is determined based on the model calibrated on data that include the misfitting persons. This calibration bias creates suboptimal conditions for person fit assessment. Solutions have been sought through the derivation of approximating bias-correction formulas and/or iterative purification procedures. Yet, here we discuss an alternative one-stage solution that involves calibrating a model expansion of the measurement model that includes a mixture component for target aberrant response patterns. A simulation study evaluates the approach under the most unfavorable and least-studied conditions for person fit indices, short polytomous survey scales, similar to those found in large-scale educational assessments such as the Program for International Student Assessment or Trends in Mathematics and Science Study.

Levine and Rubin (1979) used the term appropriateness measurement to denote methods for detecting persons with an aberrant item response pattern on a test. Aberrant is used here in the sense that the item response patterns are not corresponding to those one would typically expect to observe. Appropriateness measurement seeks to provide internal evidence indicating that the person did not approach the test as do other test-takers, and this is solely based on the item responses given, not referencing other additional information not part of the test itself (e.g., a person’s gender, religion, race, or socioeconomic status). Assessing measurement appropriateness is relevant to reach more valid test results and inferences in general, but especially to prevent making important decisions about individuals when measurement of that individual is, in fact, shown to be inappropriate (Emons, 2008).

The methodology used for the detection of aberrant item response patterns shares much in common with more general statistical approaches such as influential outlier analysis and model fit assessment, and is commonly put under the umbrella label of *person fit* in the context of item response theory (IRT). There is a wide variety in person fit statistics (for a review, see e.g., Karabatsos, 2003; Meijer & Sijtsma, 2001), including non-parametric indices such as the number of Guttman errors (Meijer, 1994) and parametric indices such as $L_{z}$ (Drasgow et al., 1985), a standardized difference between the observed and expected loglikelihood of a person’s item response pattern under an item response model of interest, given the person’s estimated latent trait. Next to more generic person fit indices, aiming to detect any type of item response pattern showing misfit, there are also more specific person fit indices that are tailored toward the detection of a target type of aberrant item response patterns. Drasgow and Levine (Drasgow & Levine, 1986; Levine & Drasgow, 1988) used the Neyman-Pearson lemma (Neyman et al., 1933) to construct optimal person fit indices (as in most powerful for detecting the target aberrant pattern) by formulating an individual likelihood ratio comparing competing models $M_{\mathrm{aberrant}}$ and $M_{\mathrm{typical}}$ for the item response pattern $y_{p}=\left(y_{p1},\ldots ,y_{pi},\ldots ,y_{\mathrm{pI}}\right)$ of a person p(p∈{1,…,n}) :

(1) $$LR\left(y_{p};M_{\mathrm{aberrant}},M_{\mathrm{typical}}\right)=\frac{l_{\mathrm{aberrant}}\left(y_{p}\right)}{l_{\mathrm{typical}}\left(y_{p}\right)}.$$

The model $M_{\mathrm{typical}}$ would be the item response model assumed to be consistent with *typical* item response patterns in the general population. The competing model $M_{\mathrm{aberrant}}$ would be consistent with the target type of *aberrant* response patterns and reflective of the type of person misfit under investigation. A participant’s response pattern is flagged as aberrant if the likelihood ratio between both models exceeds a critical value. The latter critical value can be set based on a likelihood ratio reference distribution resulting from a resampling approach (e.g., de la Torre & Deng, 2008; Sinharay, 2016; Tendeiro & Meijer, 2014).

## Sampling Variation & Built-in Calibration Bias

The performance of model-based person fit indices relies on the extent the estimated item response model $M_{\mathrm{typical}}$ is a good approximation of the true underlying data-generating model. Given that the model-based person fit indices are computed based on estimated item parameters $\hat{\beta }=\left[{\hat{\beta }}_{1},\ldots ,{\hat{\beta }}_{i},\ldots ,{\hat{\beta }}_{I}\right]$ and an individual’s estimated person parameter ${\hat{\theta }}_{p}$ under model $M_{\mathrm{typical}}$ , both sampling variation and model misspecification can be expected to take a toll (Molenaar & Hoijtink, 1990; S.E. Hong et al., 2020). With respect to sampling variation in the latent trait estimate, work by Snijders (2001) and more recent generalizations by M. Hong et al. (2021) and Gorney et al. (2024) have focused on quantifying and reducing the bias due to working with the estimated ${\hat{\theta }}_{p}$ instead of the true $\theta_{p}$ in-person fit assessment. Yet, the classical implementation of model-based person fit statistics also has a baked-in calibration bias, as the estimated model $M_{\mathrm{typical}}$ underlying the person misfit indices is calibrated on the full dataset, which includes the person under investigation, but also includes other potentially suspect individuals. Misfitting persons could potentially induce bias in the estimated model $M_{\mathrm{typical}}$ (i.e., in $\hat{\beta }$ and as a consequence in $\hat{\theta }$ ) which, in turn, further translates into bias in the discrepancy measure between the observed and expected item response pattern used to diagnose person fit. In this sense, the classic approaches can be expected to be, by definition, suboptimal, not reaching optimal detection power even when using a theoretically optimal procedure. This caveat is not unique to person fit statistics and appropriateness measurement, but is a widely known commonplace in the field of outlier detection (e.g., Rousseeuw & Leroy, 1987).

Cross-validation and other more iterative subsampling techniques might provide one pathway to consider to further optimize performance by reducing the calibration bias in the reference item response model $M_{\mathrm{typical}}$ through purification of the dataset from aberrant item response patterns and hence misfitting persons (e.g., Qiu et al., 2024). Unfortunately, this pathway promises to be cumbersome and not straightforward. Any proposed technique will naturally be computationally intensive due to the lack of closed-form formulas for the involved model expressions and diagnostics. Furthermore, it remains unclear whether the implied sequential iterative nature of the purification algorithm would have any guarantees for finding the optimal solution or even lead to a uniquely identifiable “correct” solution (for similar issues in detection of differential item functioning and model specification searches, see e.g., Candell & Drasgow, 1988; MacCallum et al., 1992; Magis & Facon, 2013).

In the absence of further developments in this direction, we propose, for specific model-based person fit indices of the likelihood ratio class, a clean alternative approach to iterative purification, and this is based on the principle of model expansion (Gelman & Shalizi, 2013) through a finite mixture of the typical item response model $M_{\mathrm{typical}}$ and the competing atypical model for aberrant item response patterns $M_{\mathrm{aberrant}}$ .

## A Mixture Model Expansion Approach

Instead of the current practice of fitting the two competing comparison models separately, each model can be incorporated as a class component model for one of two distinct latent classes in a mixture model (e.g., McLachlan et al., 2019):

$$l_{\mathrm{mixture}}\left(y_{p}\right)=\mathrm{Pr}\left(C=0\right)l_{\mathrm{typical}}\left(y_{p}|C=0\right)+\mathrm{Pr}\left(C=1\right)l_{\mathrm{aberrant}}\left(y_{p}|C=1\right)$$

where the typical identification constraints hold for the prior latent class membership probabilities, Pr(C=c)∈[0,1] and $1=\sum_{c=0}^{1}\mathrm{Pr}\left(C=c\right)$ , and the class component models— $M_{\mathrm{typical}}$ and $M_{\mathrm{aberrant}}$ —are each uniquely identified. The finite mixture model $M_{\mathrm{mixture}}$ reduces to one of its component class models when the two prior latent class membership probabilities reduce to a deterministic (0,1) combination. The consideration of a population with two unobserved groups, one group providing typical responses to the test (i.e., C=0 , consistent with $M_{\mathrm{typical}}$ ) and one group providing aberrant responses of a certain type (i.e., C=1 , consistent with $M_{\mathrm{aberrant}}$ ) is reflected by this mixture formulation. The explicit modeling of such heterogeneity in response patterns ensures that model parameters of $M_{\mathrm{typical}}$ are now implicitly adjusted for the presence of the class of persons responding in aberrant fashion, compared to when having estimated item and person parameters as part of a separate model, ignoring such potential population heterogeneity.

Instead of computing an individual likelihood ratio as a person fit index for target aberrant response patterns based on the two models being estimated separately, as in Equation 1, the logical extension in the proposed mixture model expansion would be to compare the person’s posterior class membership probabilities:

(2) $$PO\left(y_{p}\right)=\frac{\mathrm{Pr}\left(C=1|Y_{p}=y_{p}\right)}{\mathrm{Pr}\left(C=0|Y_{p}=y_{p}\right)}\;\;,$$

where PO stands for posterior odds, and the posterior class membership probabilities are computed as

$$\begin{array}{c} \mathrm{Pr}\left(C=1|Y_{p}=y_{p}\right)=\frac{l_{\mathrm{aberrant}}\left(y_{p}|C=1\right)\mathrm{Pr}\left(C=1\right)}{l_{\mathrm{mixture}}\left(y_{p}\right)}\\ \mathrm{Pr}\left(C=0|Y_{p}=y_{p}\right)=\frac{l_{\mathrm{typical}}\left(y_{p}|C=0\right)\mathrm{Pr}\left(C=0\right)}{l_{\mathrm{mixture}}\left(y_{p}\right)}\;\;. \end{array}$$

The common denominator in both terms allows simplifying the posterior odds to the product of the prior odds of class membership with the component class model likelihood ratio

$$PO\left(y_{p}\right)=\frac{\mathrm{Pr}\left(C=1\right)}{\mathrm{Pr}\left(C=0\right)}\times \frac{l_{\mathrm{aberrant}}\left(y_{p}|C=1\right)}{l_{t\mathrm{ypical}}\left(y_{p}|C=0\right)}\;\;.$$

The prior odds of class membership $\frac{\mathrm{Pr}\left(C=1\right)}{\mathrm{Pr}\left(C=0\right)}$ reflects the baseline odds of class membership in the population as estimated by the mixture model, and, for an individual person, these prior odds are then updated using what you can learn from this person’s observed response pattern $y_{p}$ , where $\frac{l_{\mathrm{aberrant}}\left(y_{p}|C=1\right)}{l_{\mathrm{typical}}\left(y_{p}|C=0\right)}$ can be seen as a type of Bayes factor.

When used for classification purposes, mixture models also rely on these posterior odds and class membership probabilities. The most common decision rule for classification assigns a person to the class for which the person has the maximum posterior class membership probability:

$$\begin{array}{c} PO\left(y_{p}\right)>1\equiv \mathrm{Pr}\left(C=1|Y_{p}=y_{p}\right)>\mathrm{Pr}\left(C=0|Y_{p}=y_{p}\right)\Rightarrow \;\mathrm{aberrant};\\ PO\left(y_{p}\right)\leq 1\equiv \mathrm{Pr}\left(C=1|Y_{p}=y_{p}\right)\leq \mathrm{Pr}\left(C=0|Y_{p}=y_{p}\right)\Rightarrow \;\mathrm{typical}. \end{array}$$

Hence, in our setting, the classification rule provides an immediate person fit assessment and measurement appropriateness decision for the person based on their observed item response pattern and the mixture model $M_{\mathrm{mixture}}$ . The estimated baseline rate in the population provides additional context information on person misfit, and the person’s individual posterior class membership probability for the atypical class is an excellent candidate for effect size for measurement inappropriateness. To further quantify the robustness of the classification, we suggest computing the minimum number of item responses that need to be changed before a person’s posterior classification would change.

### Advanced Organizer

The advantage of a mixture model expansion approach to person fit is that for targeted specific person fit indices, only one model is estimated, which on top has a built-in bias correction accounting for the potential presence of a population subgroup answering in the specified aberrant fashion to the test. By reconceptualizing the person fit question as a mixture problem, one can also work conveniently within a classification framework. In what follows, we adopt the proposed mixture model expansion approach to diagnose person misfit as caused by random item response patterns, a type of aberrant response pattern that is notoriously difficult to detect (e.g., M. Hong et al., 2020). We will also focus on ordinal responses and relatively short scales as common in educational survey research, as this is an area where interest in such measurement appropriateness monitoring is growing, but the research base is scarcer in comparison with person fit research on large achievement tests with binary responses (Rupp, 2013). The short scales setting forms a challenging context for person fit diagnostics as less information is available to distinguish between typical and aberrant responses, and hence, it is here where one would hope that any form of optimization of the person fit detection procedures, as suggested by the proposed mixture model expansion, would prove its worth.

## Implementation of Random Response Patterns

**Measurement model $M_{\mathrm{typical}}$ :** The majority of persons are expected to show a typical response pattern in correspondence with an item response measurement model, such as for instance the Graded Response Model (GRM: Samejima, 1969), where item responses of a person are conditionally independent given the person’s latent trait $\theta_{p}$

(3) $$\mathrm{Pr}\left(Y_{p}=y_{p}\right)=\int_{\theta }\underset{i}{\Pi }\mathrm{Pr}\left(Y_{pi}=y_{pi}|\theta_{p}\right)h\left(\theta \right)d\theta .$$

In the GRM, the conditional cumulative distribution function (cdf) of answering in a category k ( k=1,2,…,K ) or lower on item i given the person’s latent trait $\theta_{p}$ is written as

$$F\left(Y_{pi}=k|\theta_{p}\right)=\frac{1}{1+\exp\left(-\alpha_{i}\theta_{p}-\beta_{ik}\right)},$$

in which $\alpha_{i}$ is recognized as an item discrimination parameter and $\beta_{ik}$ as an item category threshold parameter. The item response probabilities are formed as differences between adjacent category cdfs: $\mathrm{Pr}\left(Y_{pi}=k|\theta_{p}\right)=F\left(Y_{pi}=k|\theta_{p}\right)-F\left(Y_{pi}=k-1|\theta_{p}\right)$ .

**Random model $M_{\mathrm{aberrant}}$ :** In contrast, a minority of people are expected to show aberrant response patterns in line with a uniform null model. The model can be expressed as a reduced version of the GRM. The common latent trait that previously linked responses within a person is dropped, such that item responses are now mutually independent

(4) $$\mathrm{Pr}\left(Y_{p}=y_{p}\right)=\underset{i}{\Pi }\mathrm{Pr}\left(Y_{pi}=y_{pi}\right),$$

with the cdf formulated as

$$F\left(Y_{pi}=k\right)=\frac{1}{1+\exp\left(-\gamma_{k}\right)}.$$

The item category threshold parameters are equal across items and are fixed to $\gamma_{k}=-\log\left(K/k-1\right)$ such that each response category k among the K categories has an equal uniform probability of occurrence of 1/K .

**Mixture model $M_{\mathrm{mixture}}$ :** Mixing both models $M_{\mathrm{typical}}$ and $M_{\mathrm{aberrant}}$ by postulating two underlying yet unobserved groups in the population, students engaging in typical response behavior versus students engaging in invalid random response behavior, gives rise to the mixture model. The resulting model is an extension of an instance of the HYBRID model by Yamamoto (1989) from binary responses to the polytomous case (e.g., Mislevy & Verhelst, 1990; van Laar & Braeken, 2022).

The component weight Pr(C=RR) can be interpreted as a prevalence estimate of random responders on the questionnaire scale to which the items under investigation belong. Notice that this component weight or prior latent class membership probability is also the only additional model parameter that needs to be estimated in this mixture model $M_{\mathrm{mixture}}$ as the specific component model for aberrant responses is defined only in terms of fixed parameters.

### Appropriateness Measurement

Appropriate measurement then comes down to assessing whether the person’s observed item response pattern is more in line with response patterns consistent with the uniform null model $M_{\mathrm{aberrant}}$ or more in line with response patterns consistent with the typical measurement model $M_{\mathrm{typical}}$ . The classic approach to appropriateness measurement would first fit both models $M_{\mathrm{typical}}$ and $M_{\mathrm{aberrant}}$ separately and then compute a person’s individual likelihood ratio for their response pattern under both models. The adopted uniform null model $M_{\mathrm{aberrant}}$ for the random response patterns have the advantage that it does not need to be estimated because the model’s likelihood essentially simplifies to a constant for each person’s response pattern:

$$l_{\mathrm{aberrant}}\left(y_{p}\right)=\overset{I}{\underset{i}{\Pi }}\left(1/K\right).$$

Thus, we have the following type-II index of measurement appropriateness (Levine & Rubin, 1979)

(5) $$LR\left(y_{p};M_{\mathrm{aberrant}},M_{\mathrm{typical}}\right)=\frac{l_{\mathrm{aberrant}}\left(y_{p}\right)}{l_{\mathrm{typical}}\left(y_{p}\right)}=\frac{\overset{I}{\underset{i}{\Pi }}\left(1/K\right)}{l_{\mathrm{typical}}\left(y_{p}\right)}\;\;.$$

The proposed alternative approach using a posterior odds under a mixture model expansion of both models $M_{\mathrm{typical}}$ and $M_{\mathrm{aberrant}}$ requires fitting a single model $M_{\mathrm{mixture}}$ . Where the posterior odds then simplify to

$$PO\left(y_{p};M_{\mathrm{mixture}}\right)=\frac{\mathrm{Pr}\left(C=1\right)}{\mathrm{Pr}\left(C=0\right)}\times \frac{l_{\mathrm{aberrant}}\left(y_{p}|C=1\right)}{l_{\mathrm{typical}}\left(y_{p}|C=0\right)}=\frac{\mathrm{Pr}\left(C=1\right)}{\mathrm{Pr}\left(C=0\right)}\times \frac{\overset{I}{\underset{i}{\Pi }}\left(1/K\right)}{l_{\mathrm{typical}}\left(y_{p}|C=0\right)}.$$

Both approaches share the constant reference value $\overset{I}{\underset{i}{\Pi }}\left(1/K\right)$ of $M_{\mathrm{aberrant}}$ as a reference baseline factor in the numerator of their person fit statistic. Yet, the main difference between both approaches is that the mixture-expansion approach would (a) make use of information on the estimated prevalence in the population (i.e., Pr(C=1) ) and (b) have the measurement model in $M_{\mathrm{typical}}$ corrected for the presence of random responders (i.e., $l_{\mathrm{typical}}\left(y_{p}|C=0\right)$ instead of $l_{\mathrm{typical}}\left(y_{p}\right)$ ). In contrast, the classical person fit account acts blindly to the potential presence of random response patterns as if under the working assumption that there are no such aberrant response patterns. Thus, the mixture-expansion approach should, at least in theory, have clear advantages to the classical approach to measurement appropriateness assessment. We will set up a Monte Carlo simulation experiment to put the proposed mixture-expansion approach to the test and compare the performance of both approaches.

## Simulation Study

In designing the simulation design we follow factors outlined by Rupp (2013) and Credé (2010) and are guided by the theoretical differences between the two-person fit approaches considered in our study. As a realistic context for the simulation study, we consider the typical survey scales in student questionnaires of international large-scale assessments in education (e.g., IEA: Trends in Mathematics and Science Study [TIMSS], OECD: Program for International Student Assessment [PISA]). Due to the low stakes involved for the students participating in these assessments, data quality control in the form of appropriateness measurement can have its value as it is expected that at least a minority of students will not answer the surveys fully engaged, opening up for the risk of aberrant random response patterns on some or all of the survey scales. The described context does provide generally unfavorable conditions for person fit indices, as survey scales tend to be much shorter in length than achievement tests. In less favorable conditions, any gain in accuracy as suggested by the proposed mixture-expansion approach to person fit should be welcome. In our study, we will therefore consider fixed-length scales of I=8 items with K=4 ordered categories. Given the shortness of the scales, we will also restrict the study to a setting where group membership does not change across the scale (i.e., a person is assessed as scale-level “random responder”, without singling out a response on an individual item as random).

### Prevalence of Random Responders

With respect to the prevalence of random responders, we consider 10% and 20% of random responding in the population ( RR%={10,20} ). These levels correspond to prevalence estimates found in the literature (e.g., Credé, 2010; van Laar & Braeken, 2024). Person’s group membership will be drawn probabilistically with RR% the assignment probability of being a member of the random responder group (and the complementary opposite probability for membership of the typical responder group). For the traditional individual likelihood ratio approach, person misfit detection rates tend to decrease with an increase in the number of aberrant responding persons because typical and atypical persons become harder to distinguish (Rupp, 2013). Hence, we expect the biggest performance discrepancy between the two approaches to occur for conditions with RR%=20 . To investigate the absolute type-I error rate, we also include the boundary situation where no one in the population belongs to the random responder subpopulation ( RR%=0 ). The latter conditions will also be used to determine the individual loglikelihood ratio threshold for the traditional method; this will be set at the observed 95% quantile for this statistic such that an implied 5% type-I error rate applies (Sinharay, 2016).

### Sample Size

The sample size for the dataset at hand can be expected to influence both approaches, as both are model-based and require an initial calibration of the (mixture) item response model. More data should facilitate a better calibration and hence better performance of both approaches. Two sample sizes n={1000,4000} will be considered, resembling the minimum sample size and typical sample size for data collected for a country participating in the earlier-mentioned international large-scale assessments in education. Person parameters $\theta_{p}$ for the GRM, will be drawn from a standard normal distribution (i.e., $\theta_{p}\sim N\left(0,\;1\right)$ ).

### Item Discrimination

The more consistent the typical responders’ response patterns, the easier for both approaches to identify the random responders. This feature is directly connected to the strength of the measurement model and was manipulated by varying the size of the discrimination parameters of the GRM for typical responders. For all items in the survey scale, their standardized discrimination parameter $a_{i}$ was drawn from a normal distribution with a standard deviation .05 and a mean set at one of two levels a={.4,.7} (i.e., $a_{i}\sim N\left(a,\;.05\right)$ ). The unstandardized discrimination parameters are then obtained by backtransforming to the logit scale following $\alpha_{i}=\sqrt{\frac{\pi^{2}}{3}\frac{a^{2}}{1-a^{2}}}$ .

### Item Thresholds

The tendency for responding in given categories for typical responders was manipulated by varying the set of category thresholds of items in the scale, being either skewed, modal, or uniform within an item. These threshold types corresponded to the following set of cumulative category proportions for an average typical responder (i.e., $\theta_{p}=0$ ): $p_{\mathrm{skewed}}=\{.20,.30,.50\}$ , $p_{\mathrm{modal}}=\{.15,.50,.85\}$ , and $p_{\mathrm{uniform}}=\{.25,.50,.75\}$ . These cumulative proportions were then logit-transformed to obtain standardized category thresholds $b=\{b_{1},b_{2},b_{3}\}$ and consequently put in their unstandardized form on the logit scale $\beta_{ic}=b_{ic}\sqrt{\frac{\pi^{2}}{3\left(1-a_{i}^{2}\right)}}$ for the GRM. Item category threshold parameters were set to be parallel across items within the scale. A more unequal, nonuniform use of response categories by typical responders could facilitate distinguishing them from the aberrant random responder group for both approaches.

### Study Design

**Figure 1.**
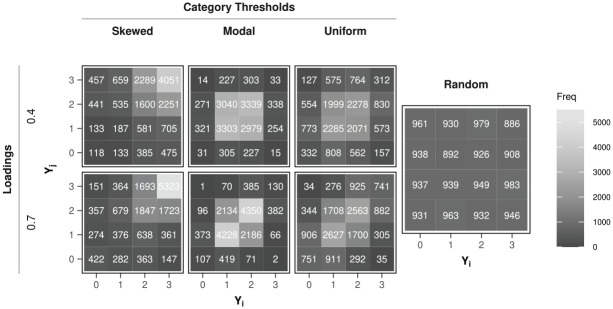
Bivariate item response distributions $Y=\left(Y_{i},Y_{j}\right)$ under model $M_{\mathrm{typical}}$ as implied by the study design (loadings by category thresholds) contrasted to the corresponding distribution under $M_{\mathrm{aberrant}}$ (i.e., Random). *Note.* Both item responses $Y_{i}$ and $Y_{j}$ have four response categories (0–3).

Together, these four experimental factors (i.e., RR%,n,a,p) combine to form a fully crossed design with 3×2×2×3=36 conditions under which both person fit approaches will be contrasted. For each condition, 100 independent replications were run. To visualize the observed response patterns that the design choices imply, Figure 1 displays the resulting bivariate item response distribution given the 2×3 item discrimination and threshold combinations under zero prevalence of random responders (i.e., model $M_{\mathrm{typical}}$ applies) and contrasts these to the bivariate response distributions one would observe if there were only random responders (i.e., 100% prevalence and model $M_{\mathrm{aberrant}}$ applies). The not-represented conditions are a mixture of these two extremes.

The sumscore distribution under $M_{\mathrm{aberrant}}$ will look rather similar to that for $M_{\mathrm{typical}}$ given modal or uniform category threshold sets for items, but the skewed threshold sets will naturally lead to a more skewed sumscore distribution. Hence, based on this observation, one might assume that the condition with skewed thresholds will facilitate making the distinction between an item response pattern more likely under $M_{\mathrm{aberrant}}$ than under $M_{\mathrm{typical}}$ . Yet, when computing the likelihood ratio statistic based on the comparison of the chi-square cross-tables of observed bivariate response patterns under $M_{\mathrm{aberrant}}$ versus $M_{\mathrm{typical}}$ it shows that the modal category thresholds produce item response patterns that are overall more distinct from those under $M_{\mathrm{aberrant}}$ , than the two other threshold sets.

### Outcome Measures

In an initial step, we will report on the convergence of the mixture model $M_{\mathrm{mixture}}$ and the extent that the data allows for a clear separation from the traditional model $M_{\mathrm{typical}}$ as indicated by a comparison of the models’ loglikelihoods. The latter comparison will be visualized using landscape plots (e.g., Braeken & Paap, 2020; Navarro et al., 2004).

#### Parameter Estimation

Subsequently, we will map the observable implications of ignoring the presence of random responders on indices relevant to the estimation of both item as well as person parameters. We will study the (averaged across items) estimation bias of item discrimination and item category threshold parameters. To assess the overall impact, we will compare the resulting test information curves across a grid for the latent trait ${\hat{\theta }}_{p}\in [-2.5,2.5]$ .

#### Classification

Finally, both the traditional and the mixture-expansion approach for appropriateness measurement will be assessed based on how they partition the sample into typical and aberrant responders. We will compute the adjusted Rand index (Hubert & Arabie, 1985) to quantify partition recovery, but also the predictive value of being flagged as aberrant, and the accuracy of the estimated prevalence of random responders. To quantify the robustness of the classification, we will compute for each person the number of responses that needed to be substituted to change their classification, that is, the minimal Hamming distance (Hamming, 1950) of their item response pattern to an observed response pattern of the opposite class^1^. For the mixture approach, we will also evaluate the strength of classification assignment as expressed by the posterior class probability.

### Data Analysis

To help form an idea about the relative importance of the variation of the different experimental factors within the context of our study design, we will compute two *R*-squared effect size measures: $R_{\mathrm{single}}^{2}$ and $\Delta R_{\mathrm{dropped}}^{2}$ . The former $R_{\mathrm{single}}^{2}$ is the percentage of variance in the outcome accounted for by the experimental factor of interest as given by a linear model where it functions as the single predictor. The latter $\Delta R_{\mathrm{dropped}}^{2}$ is the difference in percentage of variance accounted for in the outcome between the linear model reflecting the full factorial experimental design (including all higher-order terms) and that same model for which all terms related to the experimental factor of interest are dropped (i.e., first-order and higher-order terms). Large differences between the $R_{\mathrm{single}}^{2}$ and $R_{\mathrm{dropped}}^{2}$ effect size measures, in favor of the latter, are indicative of the presence of overlap or interactions between the experimental factors in the study design.

#### Statistical Software

Custom code in R (R Core Team, 2020) was written to generate data and to analyze and visualize the simulation results. Through Mplus (Muthén & Muthén, 1998–2017) and the MplusAutomation package as Mplus-R-interface (Hallquist & Wiley, 2018), models were estimated using an expectation-maximization algorithm following a marginal maximum likelihood approach with 15 quadrature points in combination with a multistart procedure for the mixture models (100 initial stage starts and 20 final stage optimizations). The discrimination parameters were constrained to be nonnegative to prevent label-switching of the poles of the latent trait scale, hereby ensuring straightforward comparison across replications.

## Results

### Convergence

In the simulation, we observed 100% convergence for the GRM without any warnings or errors. For the mixture model, a minority of eight replications in the high-loading skewed thresholds nonzero-prevalence conditions did not replicate their best loglikelihood in the given starting set, indicating that a higher number of starting sets would have been recommended to help ensure not ending up with a local maximum. These replications were excluded from further analyses. For the zero-prevalence conditions ( RR%=0 ) specifically, a minority of 10 replications led to issues connected to the prior latent class membership probability parameter being on the boundary of its parameter space (i.e., $\hat{\mathrm{Pr}\left(C=1\right)}=0$ ), making it hard to impossible to derive the parameter’s standard error. However, this did not affect the estimated model parameters, and hence, these replications were kept. Altogether, this led to an effective sample size of N=3592 of the 36×100=3600 replications run across all conditions (i.e., 99.78%).

### Model Separation & Misspecification Bias

#### Model Separation

In each of the experimental conditions, both the regular GRM $M_{\mathrm{typical}}$ and the mixture expansion $M_{\mathrm{mixture}}$ were fitted, allowing for straightforward model comparison. The pattern of results was proportionally equivalent across sample size levels n=1000 and n=4000 , and hence differences in loglikelihoods were normalized by sample size when reporting results. In conditions free of aberrant random responders RR%=0 , both models converged on the same loglikelihood value (i.e., $\Delta \left([\mathrm{LL}\left(M_{\mathrm{typical}}\;\right)-\mathrm{LL}\left(M_{\mathrm{mixture}}\right)]/n\right)\approx 0$ ). This is in line with expectations as the mixture-expansion model $M_{\mathrm{mixture}}$ reduces to $M_{\mathrm{typical}}$ when $\hat{\mathrm{Pr}\left(C=1\right)}=0$ .

In the case of nonzero random responder prevalence RR%>0 , the largest sample-size-normalized loglikelihood differences were observed in the conditions with modal item category thresholds, followed from a distance by conditions with uniform or skewed thresholds (median $\Delta \left([\mathrm{LL}\left(M_{\mathrm{typical}}\;\right)-\mathrm{LL}\left(M_{\mathrm{mixture}}\right)]/n\right)=.36,.06,\;\mathrm{and}\;.01)$ , respectively). The threshold factor accounts on its own for about $R_{\mathrm{single}}^{2}=73\%$ of the variance in the observed loglikelihood differences between the two models, and dropping the factor including its interactions with other experimental factors would lead to a dramatic reduction of $\Delta R_{\mathrm{dropped}}^{2}=83\%$ in variance accounted for. This is in line with the bivariate foreshadowing of results provided in Figure 1. Next to this dominant experimental factor formed by the threshold differences, larger loglikelihood differences, between the GRM $M_{\mathrm{typical}}$ and the mixture expansion $M_{\mathrm{mixture}}$ , were observed for conditions with higher loadings ( $R_{\mathrm{single}}^{2}=9\%$ , $\Delta R_{\mathrm{dropped}}^{2}=15\%$ ) and with higher prevalence ( $R_{\mathrm{single}}^{2}=6\%$ , $\Delta R_{\mathrm{dropped}}^{2}=11\%$ ). The latter findings were in line with theoretical expectations; having a stronger measurement model for the typical responders and more aberrant responders should lead to a more distinct separation of both subpopulations.

#### Misspecification Bias

In the conditions free of aberrant random responders RR%=0 , the sets of parameter estimates under the regular GRM $M_{\mathrm{typical}}$ and the mixture-expansion model were approximately indistinguishable (cf. $\Delta \left([\mathrm{parameter}\left(M_{\mathrm{mixture}}\right)-\mathrm{parameter}\left(M_{\mathrm{mixture}}\right)]\right)\approx 0$ ). This result follows from both models landing on the same loglikelihood value (cf. model equivalence).

In contrast, while the bias in estimated item parameters under the mixture-expansion model $M_{\mathrm{mixture}}$ remained near 0% of the true parameter value, the estimated discrimination parameters under the GRM $M_{\mathrm{typical}}$ were, on average, across all nonzero-prevalence conditions, underestimated by about 13% and the item category thresholds were biased by about 7% toward a more balanced marginal distribution across categories. Sample size n differences among conditions did not coincide with appreciable differences in bias, yet not surprisingly, slightly higher variation in bias could be observed across replications in the lower sample size n=1000 conditions compared to the higher sample size n=4000 conditions.

##### Item Discrimination

**Figure 2.**
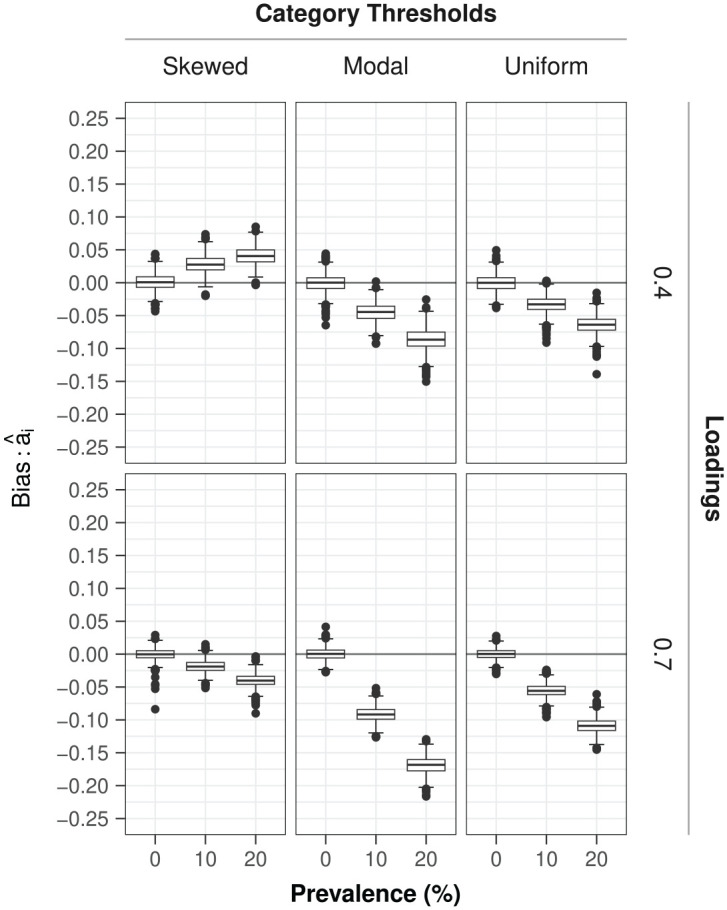
Estimation bias in the standardized item discrimination parameters (loadings) $a_{i}$ under model $M_{\mathrm{typical}}$ in the presence of varying prevalence levels of random responders in the study design. *Note.* Bias is defined as *${\hat{a}}_{i}-a_{i}^{\left(\mathrm{true}\right)}$* such that positive/negative values correspond to over/underestimation of the true parameter value by its sample estimate.

The above-mentioned bias in the discrimination parameters $\alpha_{i}$ due to ignoring random responders was larger for conditions with higher prevalence rates ( $R_{\mathrm{single}}^{2}=28\%$ , $R_{\mathrm{dropped}}^{2}=53\%$ ), for higher ( a=.7 ) versus lower ( a=.4 ) factor loadings ( $R_{\mathrm{single}}^{2}=22\%$ , $R_{\mathrm{dropped}}^{2}=37\%$ ), and for modal item category thresholds (versus the other two types) ( $R_{\mathrm{single}}^{2}=19\%$ , $R_{\mathrm{dropped}}^{2}=31\%$ ). The bias for nonzero-prevalence conditions amounted to an average underestimation of the discrimination parameter of about .07 on a standardized scale (i.e., in loading $a_{i}$ not discrimination in $\alpha_{i}$ ) (see Figure 2); Notice that the conditions with the combination of items with a skewed set of category thresholds and low loadings formed the exception to the rule.

##### Item Category Thresholds

**Figure 3.**
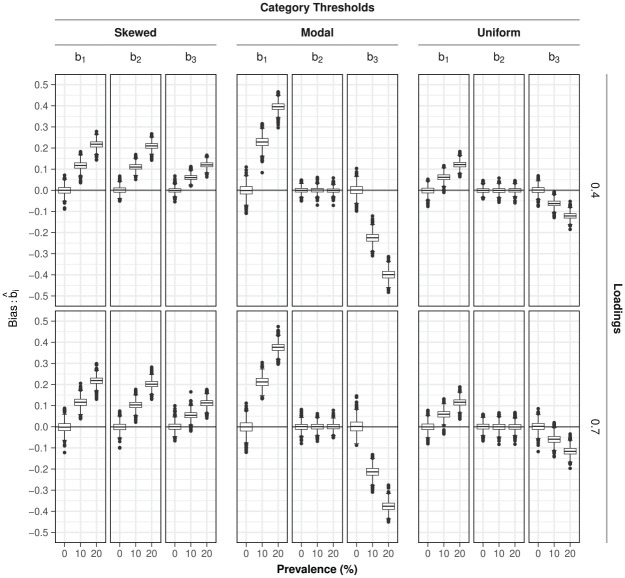
Estimation bias in the standardized item category thresholds $b_{ic}$ under model $M_{\mathrm{typical}}$ in the presence of varying prevalence levels of random responders in the study design. *Note.* Bias is defined as ${\hat{b}}_{i}-b_{i}^{\left(\mathrm{true}\right)}$ such that positive/negative values correspond to over/underestimation of the true parameter value by its sample estimate.

The bias in the item category thresholds $\beta_{ic}$ due to ignoring random responders was slightly more pronounced in conditions with higher ( a=.7 ) compared to lower ( a=.4 ) factor loadings ( $R_{\mathrm{single}}^{2}=\{8,1,3\}\%$ , $R_{\mathrm{dropped}}^{2}=\{14,6,9\}\%$ , for $\left\{\beta_{i1},\beta_{i2},\beta_{i3}\right\}$ , respectively) and also noticeably larger in conditions with higher prevalence rates ( $R_{\mathrm{single}}^{2}=\{53,14,17\}\%$ , $R_{\mathrm{dropped}}^{2}=\{69,46,47\}\%$ ) (see Figure 3). Substantial bias differences were also observed between conditions with different threshold types ( $R_{\mathrm{single}}^{2}=\{20,44,45\}\%$ , $R_{\mathrm{dropped}}^{2}=\{33,78,76\}\%$ ). For conditions with items characterized by modal category thresholds, the estimated outer threshold parameters (i.e., $\beta_{i1}$ and $\beta_{i3}$ ) were shrunk toward zero; the same pattern was observed for those with uniform category thresholds, but with less large bias; whereas for conditions with skewed category thresholds, all thresholds were overestimated ( $\beta_{i1}$ and $\beta_{i2}$ more than $\beta_{i3}$ ).

Although seemingly complex, the described trends in the bias simply reflect that the estimated GRM needed to accommodate the more balanced use of response categories due to the presence of random responders compared to what would be expected under the true GRM (i.e., category response curves are moved out of the extremes and spread out more evenly). This is further illustrated in Table 1, where the implied marginal cdf of an item response is shown under the GRM assuming a normally distributed latent trait and using the true versus estimated item parameters.

**Table 1.** Implied Marginal Cumulative Distribution Function of an Item Response Under the Graded Response Model with True Versus Estimated Item Parameters.

Parameter	Category thresholds								
	Skewed			Modal			Uniform		
	c=1	c=2	c=3	c=1	c=2	c=3	c=1	c=2	c=3
$\beta_{ic}$	−3.52	−2.15	0.00	−4.41	0.00	4.41	−2.79	0.00	2.79
${\hat{\beta }}_{ic}$	−2.82	−1.56	0.27	−2.92	0.00	2.92	−2.21	0.00	2.21
$\mathrm{cdf}\left(Y_{i}=c\right)$	0.08	0.19	0.50	0.04	0.50	0.96	0.13	0.50	0.87
$\hat{\mathrm{cdf}}\left(Y_{i}=c\right)$	0.12	0.25	0.55	0.08	0.50	0.92	0.16	0.50	0.85

*Note.* Loadings $a_{i}=.7$ , corresponding to a true item discrimination of $\alpha_{i}=1.78$ and estimated item discrimination of 1.59, 1.13, and 1.33 given skewed, modal, and uniform category thresholds, respectively.

##### Test Information

To further summarize the impact of the biased item parameters due to ignoring the presence of random responders, the ratio of the implied test information curve to its true counterpart was computed across a θ=[−2.5,2.5] grid for the latent trait. The R-squared effect size measures pointed at the prevalence rate ( $R_{\mathrm{single}}^{2}=19\%$ , $R_{\mathrm{dropped}}^{2}=47\%$ ) and item category threshold type ( $R_{\mathrm{single}}^{2}=34\%$ , $R_{\mathrm{dropped}}^{2}=64\%$ ) as key factors. In conditions with modal or uniform item category thresholds, the test information under the estimated GRM $M_{\mathrm{typical}}$ was smaller compared to under the GRM with the true item parameters; the reduction was larger given higher prevalence and higher loadings, down to on average about 46% and 58% of the true test information, respectively. This implies that measurement precision is impaired in these conditions when one ignores the presence of random responders. However, with skewed item category thresholds, the test information was overestimated for conditions with low factor loadings, whereas with high factor loadings, the test information was overestimated for high latent trait values and underestimated for low latent trait values. This implies that, when one ignores the presence of random responders, measurement precision appeared artificially high in these conditions, mainly for persons scoring high on the scale.

### Accuracy of the Estimated Prevalence of Random Responders

**Figure 4.**
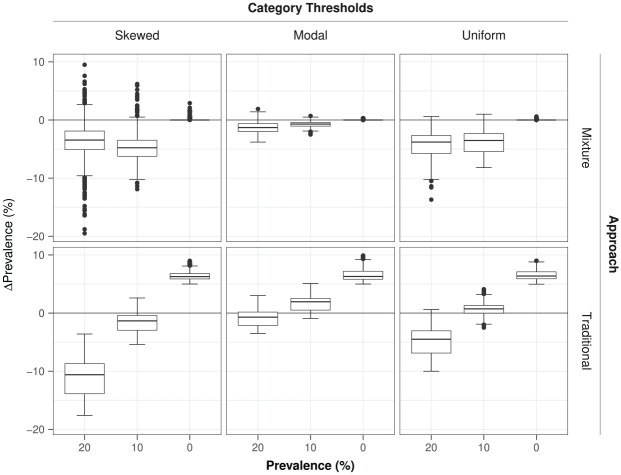
Accuracy of the estimated prevalence of random responders. *Note.* Δ Prevalence is the estimation bias in a replication defined as the difference in the percentage of persons classified as random responders with the percentage of true random responders.

The accuracy of the estimated prevalence was mostly determined by a combination of two factors, estimation approach ( $R_{\mathrm{single}}^{2}=7\%$ , $R_{\mathrm{dropped}}^{2}=29\%$ ) and prevalence ( $R_{\mathrm{single}}^{2}=46\%$ , $R_{\mathrm{dropped}}^{2}=73\%$ ), and to a lesser extent by item characteristics such as loading strength ( $R_{\mathrm{single}}^{2}=2\%$ , $R_{\mathrm{dropped}}^{2}=5\%$ ) or item category threshold type of the item parameters ( $R_{\mathrm{single}}^{2}=8\%$ , $R_{\mathrm{dropped}}^{2}=19\%$ ). When considering the estimation of the prevalence of random responders in a dataset with a small number of items, there could always be a chance that the item response pattern of a random responder might in fact end up being more consistent with the measurement model than with the random response model, hence we expected a small underestimation bias in the prevalence of random responders. This was confirmed for the mixture-expansion approach, where prevalence was underestimated by about 3% in nonzero-prevalence conditions (and 0% in zero-prevalence conditions) (see Figure 4). The traditional individual likelihood ratio approach showed a different pattern, going from a 6% overestimation in zero-prevalence conditions to a 6% underestimation at higher prevalence conditions. With its estimation bias being generally higher and a direct function of the true population prevalence, the traditional approach appeared less suitable for the estimation of the prevalence of random responders.

### Evaluation of the Classification Accuracy of the Person Fit Indices

By implication of the observed differences in estimated prevalence (i.e., aggregate level) between both approaches, differences at the individual level in classification performance were anticipated. In conditions free of aberrant random responders RR%=0 , the mixture-expansion approach using the maximum posterior class membership assignment, correctly classified all persons to be of the nonrandom responder class in the majority of replications (90%) in all zero-prevalence conditions (for the remaining replications, classification error was at most 2.5%). In contrast, the individual-level likelihood ratio tests had a false positive rate of 6%, ranging from 5% to 10% across replications, and hence only slightly more liberal than the 5% aimed for in the operationalization of the critical value by means of the Monte Carlo reference distribution.

#### Positive Predictive Value

**Figure 5.**
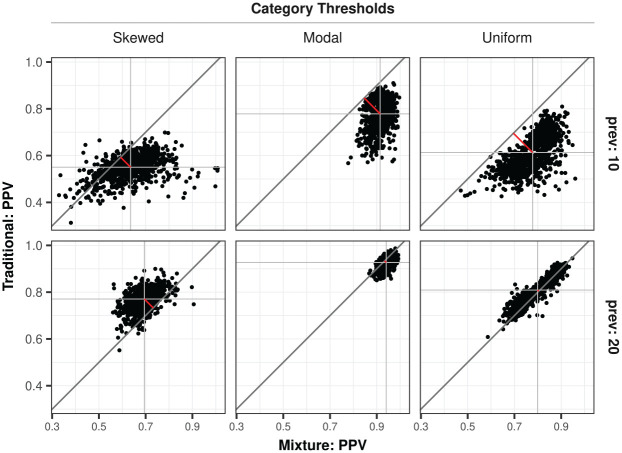
Positive predictive value of being a true random responder when flagged as such by the person fit approaches. *Note.* prev denotes the prevalence of true random responders: 10% or 20%. Each dot represents one replication in the simulation experiment.

The most relevant for the use of person fit indices in screening practices is the positive predictive value (PPV) of having been classified as a random responder, that is, the probability of being a true random responder when being flagged as such. Across all conditions with nonzero prevalence of random responders, the largest differences in PPV (see Figure 5) were observed due to differences in item category threshold type ( $R_{\mathrm{single}}^{2}=51\%$ , $R_{\mathrm{dropped}}^{2}=56\%$ )—with the skewed type providing the most challenging conditions—followed by prevalence differences ( $R_{\mathrm{single}}^{2}=17\%$ , $R_{\mathrm{dropped}}^{2}=26\%$ ), and minor differences due to loadings ( $R_{\mathrm{single}}^{2}=4\%$ , $R_{\mathrm{dropped}}^{2}=8\%$ ). The average predictive value for the mixture-expansion approach was 80% and slightly higher than the corresponding 75% for the more traditional individual likelihood ratio approach ( $R_{\mathrm{single}}^{2}=3\%$ , $R_{\mathrm{dropped}}^{2}=14\%$ ). These PPVs can be considered sufficiently high in an academic research context and for conducting sensitivity analyses for general inferences, but do not warrant stand-alone use for high-stakes individual decision-making in operational practice. Notice that the predictive value of the traditional approach is somewhat impaired under lower prevalence conditions compared to the mixture-expansion approach (i.e., on average 65% vs. 78%), but more on par around 80% under higher prevalence conditions.

Note that the counterpart, the negative predictive value (NPV) of not having been classified as a random responder, that is, the probability of being a true regular responder when not flagged, was on average a solid 94% for both person fit approaches ( $R_{\mathrm{single}}^{2}=0\%$ , $R_{\mathrm{dropped}}^{2}=6\%$ ) across all nonzero-prevalence conditions. The NPV dropped to a low of 84% in the condition with skewed thresholds, higher prevalence low loadings, whereas in all other cells of the study design, it did not go lower than 88%. Note that the high NPVs are also in line with the low baseline levels of prevalence in our study design (i.e., 80%–90% of the population should not be flagged).

#### Strength of Assignment

For the mixture-expansion approach, the persons flagged as random responders had class membership probabilities close to 1 ( $\mathrm{Pr}\left(C=1|y_{p}\right)>.97$ ) in the modal thresholds conditions versus only .64 ( $a_{i}=.4$ ) to .70 ( $a_{i}=.7$ ) in skewed thresholds conditions and .72 ( $a_{i}=.4$ ) to .89 ( $a_{i}=.7$ ) in uniform threshold conditions. Persons not flagged had a class membership probability close to zero ( $\mathrm{Pr}\left(C=1|y_{p}\right)<.03$ ) in all conditions. On average, 10% of the persons currently not flagged as random responders would receive the opposite classification upon changing only one item response, whereas for the majority, two or more responses out of the eight items in total would have to change before they would have been flagged. These results imply that the strength and robustness of assignment to the aberrant responder class are fairly solid.

## Discussion

This study proposed a mixture-expansion approach to measurement appropriateness assessment. By having a model that simultaneously mixes the reference item response model $M_{\mathrm{typical}}$ and the aberrant response model $M_{\mathrm{aberrant}}$ , the approach avoids the baked-in calibration bias of the more traditional specific person fit indices originally introduced by Drasgow and Levine (Drasgow & Levine, 1986; Levine & Drasgow, 1988). Item parameters of $M_{\mathrm{typical}}$ , the reference for typical responders is now estimated, accounting for the potential presence of aberrant responders. The proposed approach is attractive and straightforward to implement. Even in general suboptimal and understudied conditions for person fit assessment—short scales consisting of polytomous items commonly used in the student questionnaires of international large-scale assessments such as PISA and TIMSS—the approach delivers on its promise of bias-correction for item parameters in $M_{\mathrm{typical}}$ , good estimation of overall prevalence of aberrant item responses, and acceptable positive predictive validity for screening purposes.

Despite working with biased item parameters, as a result of estimating the reference model $M_{\mathrm{typical}}$ from samples that include substantial proportions of aberrant item response patterns, the traditional approach still managed to achieve reasonable PPV. The mixture expansion did improve performance with an average PPV of 79% compared to 74% for the traditional approach. These PPVs can be considered sufficiently high in an academic research context and for conducting sensitivity analyses for general inference, but do not warrant stand-alone use for high-stakes individual decision-making in operational practice.

For prevalence estimation of aberrant response patterns, the size and direction of the estimation bias in the traditional approach varied as a function of the true population variance, whereas the mixture-expansion approach displayed a slight underestimation bias in line with theoretical expectations. Therefore, the more predictable and conservative nature of the mixture-expansion approach would make it the method of choice when prevalence estimation is concerned.

A challenge in the traditional person fit approach is setting an appropriate decision threshold. In our study, the threshold is based on models estimated on data arising from comparable conditions to the dataset at hand, but now guaranteed not to contain random responders. In real-life practice, such a guarantee is not present, but one would either use a rule of thumb or use a parametric bootstrap cross-fitting procedure to suggest a reasonable empirical threshold. Yet note that the parametric bootstrap would be based on the model with baked-in calibration bias, as it is based on the original sample that potentially includes aberrant responders. Hence, one additional advantage of the mixture-expansion approach is that the threshold is model-based and guided by the empirical separation evidence between the two alternatives, typical or aberrant, for each individual item response pattern (c.f., posterior class membership probabilities). In principle, all person fit approaches can be further tweaked by augmenting their classification decision rule with a region of indecision, a gray zone, where one would conclude that the empirical evidence is not strong enough for making a solid conclusion. This would lead to a third classification outcome, “undecided” next to “aberrant” and “typical”. Such an approach can improve the predictive value of being classified as “aberrant” at the obvious cost of having to decide on what to do with those in the gray zone. When gathering data in real time, this could also be implemented as part of a monitoring procedure (see e.g., sequential probability ratio test in the context of decision theory and adaptive classification testing [Eggen, 1999; Wald, 1947]). However, even in the challenging case of short scales of 8 polytomous items, strength of assignment was already quite large (i.e., high posterior class probabilities and median Hamming distance of 2 out of 8 item responses), indicating that high discriminability between typical and aberrant item response patterns can be reached even within a short-scale setting.

Another feature of the mixture-expansion approach is that it allows for easily interpretable effect size measures in the context of person fit assessment. The posterior probability of assignment to the aberrant class $\mathrm{Pr}\left(C=1|y_{p}\right)$ provides a quite natural metric for interpretation at the individual level. Its counterpart at the population level, the estimated prior class probability Pr(C=1) , can be seen as the estimated prevalence of aberrant item response patterns in the population and as a general person fit measure reflecting the percentage of persons for which the model does not fit (cf. persons as effect sizes [Grice et al., 2020; Rudas et al., 1994]).

For the specific case of random responders, because the aberrant model $M_{\mathrm{aberrant}}$ likelihood is a constant across individuals; the individual likelihood ratio person fit statistic is equivalent to the individual model $M_{\mathrm{typical}}$ likelihood and to the popular $L_{z}$ statistic. This shows that by construction $L_{z}$ is therefore most sensitive to random response patterns, and less for other aberrant response patterns such as straightlining, which explains typical sensitivity findings in simulation studies (e.g., Jin, et al., 2018). Furthermore, traditional person fit statistics such as $L_{z}$ are not easily modified to also capture more types of aberrant response patterns, whereas a mixture-expansion approach more easily generalizes to other types of aberrant response patterns (for straightlining, see e.g., Jin et al., 2018). Note that one should work with well-defined, confirmatory aberrant mixture component models, to avoid ending up in the mixture conundrum that the classes end up being uninterpretable and can be qualified as mere artifacts picking up on undefined misfit or assumption violations of model $M_{\mathrm{typical}}$ (Bauer & Curran, 2004).

## Conclusion: Cautionary Note & Prospects

One is easily inclined to ascribe the lack of measurement appropriateness for a person to unobservable underlying reasons for providing aberrant responses. Examples of such reasons are legio: insufficient effort, carelessness, lack of engagement, reading difficulties, …; all plausible reasons that might play a role in the observation of aberrant response patterns for persons on low-stakes surveys, as in, for instance, student questionnaires as seen in international large-scale educational assessments such as TIMSS and PISA. Yet, we want to emphasize that the actual measurement appropriateness assessment is fairly neutral and does not assume any specific underlying reason: the observed response pattern is more consistent with a typical responder than with an atypical responder, or vice versa, regardless of a person’s unobserved intentions. To gain more insight into the underlying cognitive processes at play, multiple sources of information should be brought in. Meijer et al. (2008), for instance, followed through on person fit results by using observations and teacher interviews on the children flagged as aberrant responders. With the increased computerization of test and questionnaire batteries, the respondent history across scales and forms of process data, such as response times and key strokes, should also become more routinely available (for the use of screen time, see e.g., Ulitzsch et al., 2022). We anticipate that future work in the area of person fit and measurement appropriateness assessment will focus on how to integrate all these multimodal data sources, in both the modeling approach and the decision rule used. Hence, to paraphrase Drasgow et al. (1991), the future of person fit assessment and “increasing detection rates on short tests does not lie in developing even better appropriateness indices for individual tests”, but in effectively and efficiently using the new multimodal data sources that will become more routinely available. A mixture-model expansion approach will also be here, a more natural platform, than more traditional single-model indices, for further developments in person fit assessment.

## Notes

1. Note that we opted to compare to the observed response patterns, and not all $K^{I}=4^{8}$ theoretically possible response patterns, to keep the computational burden at bay.

## References

[bibr1-00131644251364252] Bauer D. J. Curran P. J. (2004). The integration of continuous and discrete latent variable models: Potential problems and promising opportunities. Psychological Methods, 9(1), 3–29. 10.1037/1082-989X.9.1.3 15053717

[bibr2-00131644251364252] Braeken J. Paap M. C. S. (2020). Making fixed-precision between-item multidimensional computerized adaptive tests even shorter by reducing the asymmetry between selection and stopping rules. Applied Psychological Measurement, 44(7–8), 531–547. 10.1177/0146621620932666 34393302 PMC7495795

[bibr3-00131644251364252] Candell G. L. Drasgow F. (1988). An iterative procedure for linking metrics and assessing item bias in item response theory. Applied Psychological Measurement, 12(3), 253–260. 10.1177/014662168801200304

[bibr4-00131644251364252] Credé M. (2010). Random responding as a threat to the validity of effect size estimates in correlational research. Educational and Psychological Measurement, 70(4), 596–612. 10.1177/0013164410366686

[bibr5-00131644251364252] de la Torre J. Deng W. (2008). Improving person-fit assessment by correcting the ability estimate and its reference distribution. Journal of Educational Measurement, 45(2), 159–177. 10.1111/j.1745-3984.2008.00058.x

[bibr6-00131644251364252] Drasgow F. Levine M. V. (1986). Optimal detection of certain forms of inappropriate test scores. Applied Psychological Measurement, 10(1), 59–67. 10.1177/014662168601000105

[bibr7-00131644251364252] Drasgow F. Levine M. V. McLaughlin M. (1991). Appropriateness measurement for some multidimensional test batteries. Applied Psychological Measurement, 15(2), 171–191. 10.1177/014662169101500207

[bibr8-00131644251364252] Drasgow F. Levine M. V. Williams E. A. (1985). Appropriateness measurement with polychotomous item response models and standardized indices. British Journal of Mathematical and Statistical Psychology, 38(1), 67–86. 10.1111/j.2044-8317.1985.tb00817.x

[bibr9-00131644251364252] Eggen T. J. H. M . (1999). Item selection in adaptive testing with the sequential probability ratio test. Applied Psychological Measurement, 23(3), 249–261. 10.1177/01466219922031365

[bibr10-00131644251364252] Emons W. H. M. (2008). Nonparametric person-fit analysis of polytomous item scores. Applied Psychological Measurement, 32(3), 224–247. 10.1177/0146621607302479

[bibr11-00131644251364252] Gelman A. Shalizi C. R. (2013). Philosophy and the practice of Bayesian statistics. British Journal of Mathematical and Statistical Psychology, 66(1), 8–38. 10.1111/j.2044-8317.2011.02037.x 22364575 PMC4476974

[bibr12-00131644251364252] Gorney K. Sinharay S. Eckerly C. (2024). Efficient corrections for standardized person-fit statistics. Psychometrika 89(2), 569–591. 10.1007/s11336-024-09960-x 38558053

[bibr13-00131644251364252] Grice J. W. Medellin E. Jones I. Horvath S. McDaniel H. O’lansen C. Baker M. (2020). Persons as effect sizes. Advances in Methods and Practices in Psychological Science, 3(4), 443–455. 10.1177/2515245920922982

[bibr14-00131644251364252] Hallquist M. N. Wiley J. F. (2018). MplusAutomation: An R package for facilitating large-scale latent variable analyses in Mplus. Structural Equation Modeling: A Multidisciplinary Journal, 25(4), 621–638. 30083048 10.1080/10705511.2017.1402334 PMC6075832

[bibr15-00131644251364252] Hamming R. W. (1950). Error detecting and error correcting codes. The Bell System Technical Journal, 29(2), 147–160. 10.1002/j.1538-7305.1950.tb00463.x

[bibr16-00131644251364252] Hong M. Lin L. Cheng Y. (2021). Asymptotically corrected person fit statistics for multidimensional constructs with simple structure and mixed item types. Psychometrika, 86(2), 464–488. 10.1007/s11336-021-09756-3 33797016

[bibr17-00131644251364252] Hong M. Steedle J. T. Cheng Y. (2020). Methods of detecting insufficient effort responding: Comparisons and practical recommendations. Educational and Psychological Measurement, 80(2), 312–345. 10.1177/0013164419865316 32158024 PMC7047258

[bibr18-00131644251364252] Hong S. E. Monroe S. Falk C. F. (2020). Performance of person-fit statistics under model misspecification. Journal of Educational Measurement, 57(3), 423–442. 10.1111/jedm.12207

[bibr19-00131644251364252] Hubert L. Arabie P. (1985). Comparing partitions. Journal of Classification, 2, 193–218. 10.1007/BF01908075

[bibr20-00131644251364252] Jin K.-Y. Chen H.-F. Wang W.-C. (2018). Mixture item response models for inattentive responding behavior. Organizational Research Methods, 21(1), 197–225. 10.1177/1094428117725792

[bibr21-00131644251364252] Karabatsos G. (2003). Comparing the aberrant response detection performance of thirty-six person-fit statistics. Applied Measurement in Education, 16(4), 277–298. 10.1207/S15324818AME1604_2

[bibr22-00131644251364252] Levine M. V. Drasgow F. (1988). Optimal appropriateness measurement. Psychometrika, 53(2), 161–176. 10.1007/BF02294130

[bibr23-00131644251364252] Levine M. V. Rubin D. B. (1979). Measuring the appropriateness of multiple-choice test scores. Journal of Educational Statistics, 4(4), 269–290. 10.2307/1164595

[bibr24-00131644251364252] MacCallum R. C. Roznowski M. Necowitz L. B. (1992). Model modifications in covariance structure analysis: The problem of capitalization on chance. Psychological Bulletin, 111(3), 490. 10.1037/0033-2909.111.3.490 16250105

[bibr25-00131644251364252] Magis D. Facon B. (2013). Item purification does not always improve DIF detection: A counterexample with Angoff’s Delta plot. Educational and Psychological Measurement, 73(2), 293–311. 10.1177/0013164412451903

[bibr26-00131644251364252] McLachlan G. J. Lee S. X. Rathnayake S. I. (2019). Finite mixture models. Annual Review of Statistics and Its Application, 6(1), 355–378. 10.1146/annurev-statistics-031017-100325

[bibr27-00131644251364252] Meijer R. R. (1994). The number of Guttman errors as a simple and powerful person-fit statistic. Applied Psychological Measurement, 18(4), 311–314. 10.1177/014662169401800402

[bibr28-00131644251364252] Meijer R. R. Egberink I. J. L. Emons W. H. M. Sijtsma K. (2008). Detection and validation of unscalable item score patterns using item response theory: An illustration with Harter’s self-perception profile for children. Journal of Personality Assessment, 90(3), 227–238. 10.1080/00223890701884921 18444119

[bibr29-00131644251364252] Meijer R. R. Sijtsma K. (2001). Methodology review: Evaluating person fit. Applied Psychological Measurement, 25(2), 107–135. 10.1177/01466210122031957

[bibr30-00131644251364252] Mislevy R. Verhelst N. (1990). Modeling item responses when different subjects employ different solution strategies. Psychometrika, 55(2), 195–215. 10.1007/BF02295283

[bibr31-00131644251364252] Molenaar I. Hoijtink H. (1990). The many null distributions of person fit indices. Psychometrika, 55, 75–106. 10.1007/BF02294745

[bibr32-00131644251364252] Muthén L. K. Muthén B. O. (1998–2017). Mplus User’s Guide. Eight Edition. Muthén & Muthén.

[bibr33-00131644251364252] Navarro D. Pitt M. Myung I. J. (2004). Assessing the distinguishability of models and the informativeness of data. Cognitive Psychology, 49(1), 47–84. 10.1016/j.cogpsych.2003.11.001 15193972

[bibr34-00131644251364252] Neyman J. Pearson E. S. Pearson K. (1933). IX. On the problem of the most efficient tests of statistical hypotheses. Philosophical Transactions of the Royal Society of London. Series A, Containing Papers of a Mathematical or Physical Character, 231(694), 289–337. 10.1098/rsta.1933.0009

[bibr35-00131644251364252] Qiu X. Huang S. Y. Wang W. C. Wang Y. G. (2024). An iterative scale purification procedure on l_z_ for the detection of aberrant responses. Multivariate Behavioral Research, 59(1), 62–77. 10.1080/00273171.2023.2211564 37261427

[bibr36-00131644251364252] R Core Team. (2020). R: A language and environment for statistical computing. R Foundation for Statistical Computing.

[bibr37-00131644251364252] Rousseeuw P. Leroy A. (1987). Robust regression and outlier detection. John Wiley & Sons.

[bibr38-00131644251364252] Rudas T. Clogg C. C. Lindsay B. G. (1994). A new index of fit based on mixture methods for the analysis of contingency tables. Journal of the Royal Statistical Society: Series B (Methodological), 56(4), 623–639. 10.1111/j.2517-6161.1994.tb02004.x

[bibr39-00131644251364252] Rupp A. A. (2013). A systematic review of the methodology for person fit research in item response theory: Lessons about generalizability of inferences from the design of simulation studies. Psychological Test and Assessment Modeling, 55(1), 3–8.

[bibr40-00131644251364252] Samejima F. (1969). Estimation of latent ability using a response pattern of graded scores. Psychometrika, 34(1), 1–97. 10.1007/BF03372160

[bibr41-00131644251364252] Sinharay S. (2016). Assessment of person fit using resampling-based approaches. Journal of Educational Measurement, 53(1), 63–85. 10.1111/jedm.12101

[bibr42-00131644251364252] Snijders T. A. B. (2001). Asymptotic null distribution of person fit statistics with estimated person parameter. Psychometrika, 66(3), 331–342. 10.1007/BF02294437

[bibr43-00131644251364252] Tendeiro J. N. Meijer R. R. (2014). Detection of invalid test scores: The usefulness of simple nonparametric statistics. Journal of Educational Measurement, 51(3), 239–259. 10.1111/jedm.12046

[bibr44-00131644251364252] Ulitzsch E. Pohl S. Khorramdel L. Kroehne U. von Davier M. (2022). A response-time-based latent response mixture model for identifying and modeling careless and insufficient effort responding in survey data. Psychometrika, 87, 593–619. 10.1007/s11336-021-09817-7 34855118 PMC9166878

[bibr45-00131644251364252] van Laar S. Braeken J . (2022). Random responders in the TIMSS 2015 student questionnaire: A threat to validity? Journal of Educational Measurement, 59(4), 470–501. 10.1111/jedm.12317

[bibr46-00131644251364252] van Laar S. Braeken J . (2024). Prevalence of random responders as a function of scale position and questionnaire length in the TIMSS 2015 eighth-grade student questionnaire. International Journal of Testing, 24(1), 24–52. 10.1080/15305058.2023.2263206

[bibr47-00131644251364252] Wald A. (1947). Sequential analysis. New York, NY: J. Wiley & Sons.

[bibr48-00131644251364252] Yamamoto K. (1989). Hybrid model of IRT and latent class models. ETS Research Report Series, RR-89-41. 10.1002/j.2333-8504.1982.tb01326.x

